# Mutagenesis of UDP‐xylose epimerase and xylan arabinosyl‐transferase decreases arabinose content and improves saccharification of rice straw

**DOI:** 10.1111/pbi.13552

**Published:** 2021-02-05

**Authors:** Chen Chen, Xianhai Zhao, Xuchuan Wang, Bo Wang, Huiling Li, Jiaxun Feng, Aimin Wu

**Affiliations:** ^1^ State Key Laboratory for Conservation and Utilization of Subtropical Agro‐bioresources Guangzhou China; ^2^ Guangdong Key Laboratory for Innovative Development and Utilization of Forest Plant Germplasm College of Forestry and Landscape Architectures South China Agricultural University Guangzhou China; ^3^ College of Agriculture South China Agricultural University Guangzhou China; ^4^ State Key Laboratory for Conservation and Utilization of Subtropical Agro‐bioresources Guangxi Research Center for Microbial and Enzyme Engineering Technology College of Life Science and Technology Guangxi University Nanning China; ^5^ Guangdong Laboratory of Lingnan Modern Agriculture Guangzhou China

**Keywords:** rice, cell wall, CRISPR, Cas9, *OsUXE*, *OsXAT*, biomass feedstock

Rice straw is an important renewable biomass resource. However, due to its slow degradation returning to the field, and the environmental pollution of conventional straw burning, it is highly desired for more efficient utilization of rice straw resource. The complex structure of rice cell wall composition impedes the efficient utilization of straw biomass. Developing new strategies to improve rice straw utilization efficiency is attractive. Glucuronoarabinoxylan (GAX) and arabinoxylan (AX) are the common types of xylan in rice, which have β1,4‐xylose backbone modified with arabinose (Ara) and/or glucuronic acid side chains. The UDP‐xylose epimerase (UXE) and xylan arabinosyl‐transferase (XAT) are the major contributors to Ara side chain biosynthesis in Golgi apparatus. UXE catalyses isomerization of UDP‐Xyl to UDP‐Ara*p*, the first step of UDP‐Ara*p* biosynthesis, and Ara*p* is then transformed to Ara*f* by UDP‐arabinopyranose mutase (UAM) in cytosol. XAT catalyses arabinosyl addition to the xylan backbone to form GAX/AX. Previous reports indicate that either higher Ara content or lower ferulic acid of rice xylan side chain would increase the enzymatic hydrolysis efficiencies (Casler and Jung, 2006; Li *et al*., 2015). Meanwhile, it has also proposed an idea of reducing arabinose side chains to improve saccharification (Konishi *et al*., 2011). Thus, we hypothesize that specifically and intensively altering degree of xylan side chain substitutions might improve the utilization efficiency of rice straw. In present study, both rice *UXE* and *XAT* knockout mutants were generated using CRISPR technology; subsequently, the saccharification efficiencies of both mutant lines, compared to the wild types (WT), were explored.

We designed three CRISPR/CAS9 constructs, each targets a pair of *OsUXE* or *OsXAT* genes in rice. After obtaining transgenic rice plants, we amplified and sequenced *OsUXE* or *OsXAT* genes from 30 independently regenerated lines. Finally, double mutants, at least two alleles at different mutation, were obtained and named as *uxe1uxe2*, *uxe1uxe3* and *xat2xat3,* respectively (Figure 1a, b). Phenotypic analysis revealed that the growth and development of both *uxe* and *xat* double mutants were not obviously affected in rice (Figure 1c), which is similar to the wheat *TaXAT2* mutant (Anders *et al*., 2012) but different with *UAM* RNAi lines that show dwarf and infertile phenotypes (Konishi *et al*., 2011). Sections of stems confirmed growth of secondary cell walls in the epidermis, phloem and xylem vessels, with no significant change compared to the WT (Figure 1d). These findings indicated that cell wall morphology was not affected in the mutant lines. Previous studies revealed that disruption of *uxe* resulted in significant reduction in the amount of Ara in the cell walls of *Arabidopsis* (Burget *et al*., 2003; Sumiyoshi *et al*., 2013).Therefore, the amount of Ara in the transgenic rice cell walls was determined. Compared with the WT (4.15%), by ion chromatography measurements the arabinose content of *uxe1uxe2* and *xat2xat3* decreased to 2.19% and 2.78%, respectively (Figure 1e). By contrast, the amount of xylose and glucose did not change in any of the mutant lines, indicating that *UXE* and *XAT* mutations have no effect on the glycan backbone of GAX or AX. Why the *UXE*s mutants were still with Ara in their cell walls? One of the reasons might be due to the remaining UXE. Rice has three *OsUXE*s. Determining the total activity of UXE in the two *uxe* double mutants indeed illustrated the residual activity of UXE in those double mutants (Figure 1f). In rice, *OsXAT2* and *OsXAT3* are homologous to *TaXAT2* (Anders *et al*., 2012). The rice *xat2xat3* double mutant showed the reduction of the Ara content, which may be due to the potential redundant functions of other members of the GT61 family. Previous UAM RNAi rice line with a significant reduction in Ara content of the cell wall showed severe developmental defects (Konishi *et al*., 2011), whereas the traits of growth in our double mutants only exhibited a slight change, except seeds per ears in *xat2xat3* mutant (Figure 1g). Such discrepancy might be caused with different target genes, the rice varieties and/or growth conditions. To further confirm the decrease in Ara content in our mutants, 2D‐HSQC was used to detect the CH‐related signal peaks of hemicellulose (Figure 1h). The chemical shifts of X‐1, X‐4, X‐3, X‐2 and X‐5 of the 1, 4‐β‐D xylose unit were 101.77/4.30 ppm, 75.60/3.63 ppm, 74.09/3.36 ppm, 72.81/3.15 ppm and 63.43/3.95 ppm, respectively (Sun *et al*., 1996), and all xylose (X) signals showed almost no obvious alteration. The weak signal peak (green) from 4‐*O* methyl glucuronic acid and the unchanged structure of the xylose backbone were consistent in all samples. However, the samples of mutants showed weaker signals assigned to α‐L‐Ara in A1‐A5 (Figure 1h), indicative of reduction of Ara content. Our 2D‐HSQC results were consistent with the results of hemi‐cellulosic monosaccharide composition analysis, further confirming that Ara content was decreased in mutants.

**Figure 1:**
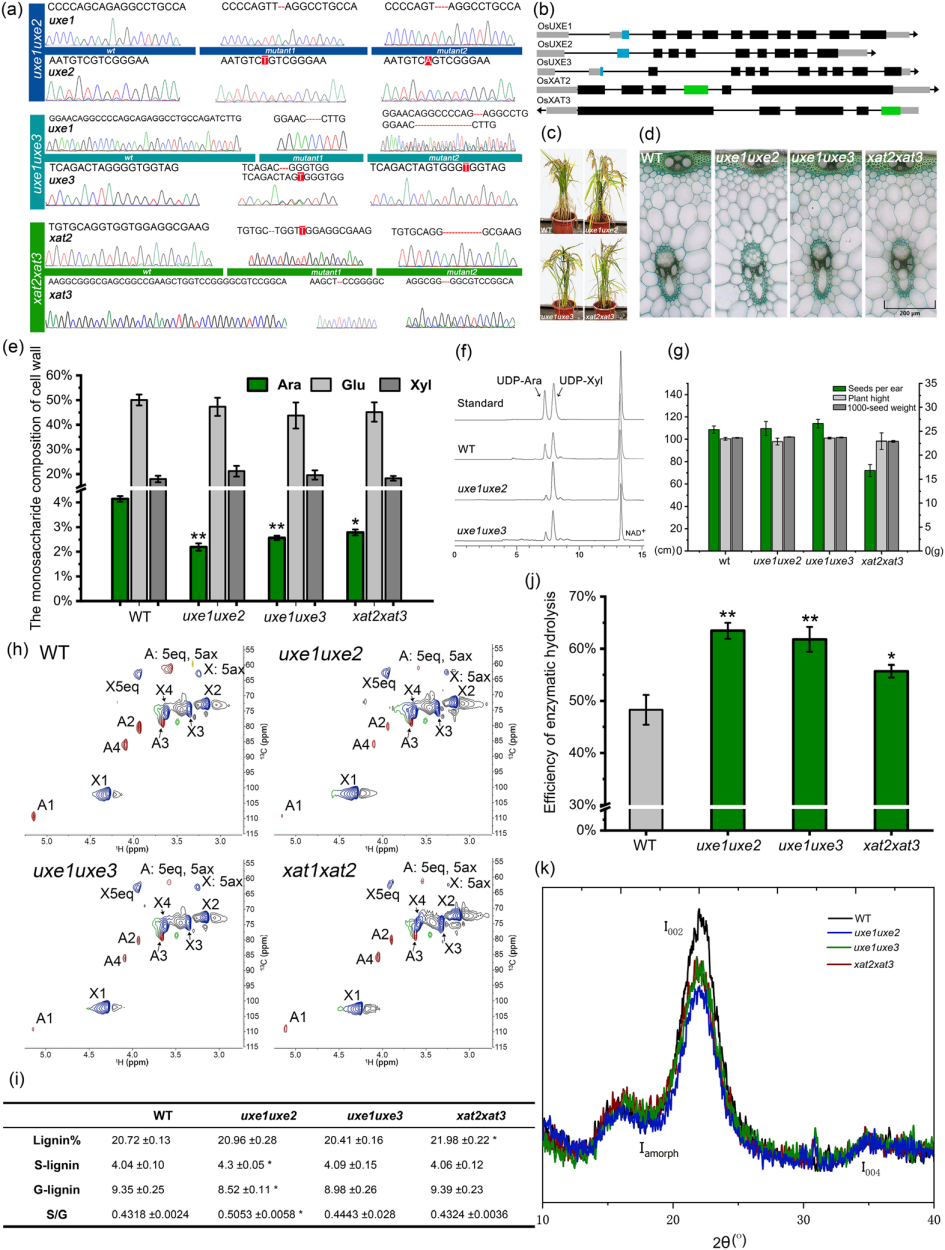
The cell wall changes of uxe and xat mutants improve the ability of straw to be hydrolysed. (a) The mutation site of the mutant (by CRISPR/Cas 9). Red markers represent mutations and dashed lines represent missing bases. (b) Schematic representation of targets in genes. Block shows exons and coloured section indicates target position. Line represents introns. (c) Photographs of plant growth. (d) Cross sectioning of rice stems. Toluidine blue dye was used. (e) Determination of amounts of arabinose, xylose and glucose in plant stem cell wall. Results are shown as ratio of monosaccharides to cell wall weight. (f) Enzyme activity of UXEs among uxes mutants and WT by extracted microsome with method referenced by Gu et al. (https://doi.org/10.1099/mic.0.040758‐0). (g) Growth condition statistics of mutants in the field. (h) 2D‐HSQC comparative of hemicellulose. Abbreviations: A, Araf; X, Xylp; (i) determination of amount of lignin of plant stem cell wall by pyGC‐MS. S (%) and G (%) data indicate proportion of syringyl units and guaiacyl units in total composition, respectively. (j) Enzymatic hydrolysis efficiency of cellulose in rice mutants. (k) XRD of uxes and xats. Scanning speed: 5°/min. Three biological replicates were used as test samples, and error bars are based on three data results. Asterisk indicates that sample is significant compared to WT (‘*’, *P* < 0.05; ‘**’, *P* < 0.01; Student's *t*‐test).

Ara is a vital component mediating linkage between xylan and lignin, because ferulic acid at the xylan Araosyl side chain links with monolignols. We used pyGC‐MS to determine the lignin content and composition. The amount of lignin in mutants was largely consistent with that of the WT plants, except for *xat2xat3* showing an increase (Figure 1i). The G‐lignin unit was decreased but S/G ratio was increased in *uxe* double mutants. Normally, not only S/G ratio, but also Ara content and G‐type lignin are significantly correlated with enzymatic saccharification (Li *et al*., 2015; Wang *et al*., 2016; Wu *et al*., 2013). So saccharification was analysed with three double‐mutant rice straw. As expected, all mutants exhibited significant increase in simple sugar release (Figure 1j). In particular, the saccharification efficiency of the *uxe1uxe2* mutants improved by 15.19%, while *xat2xat3* mutants showed an increase of 7.42%. Due to part of Ara in GAX and ferulic acid is considered to affect digestibility of cellulose (Casler and Jung, 2006), a large amount of Ara loss will loosen the cell wall structure, thereby increasing the accessibility of cellulase (Marriott *et al*., 2016; Wu *et al*., 2013).

Previous reports indicate that moderate increase of arabinose may have a negative effect on the cellulose crystallinity, which in turn affects enzymatic hydrolysis (Li *et al*., 2015). To explain our double mutants with improved saccharification efficiency, cellulose crystallinity was examined. As expected, the *uxe1uxe2, uxe1uxe3* and *xat2xat3* double mutants with less Ara showed reduced cellulose crystallinity (Figure 1k), which implies that excessive reduction of the Ara content in xylan also impairs the cellulose crystalline structures. In turn, it makes the cell walls of both *uxe* and *xat* rice mutants more hydrolysis‐prone. These results confirm our assumption that xylan with extremely low Ara side chain also has a positive effect on enzymatic hydrolysis.

In summary, knockout of *uxe* and *xat* by CRISPR / Cas9 resulted in a decrease in cell wall arabinose content. The alterations in cellulose crystallinity and enzymolysis efficiency of the mutant indicate that the cell wall structure in the mutants is loose and easier to use. We provide an environmentally friendly way of using rice straw efficiently.

## Accession number

LOC4342364 (OsUXE1), LOC4337011 (OsUXE2), LOC4344584 (OsUXE3), LOC4329205 (OsXAT2), LOC4333275 (OsXAT3).

## Conflict of interest

No conflict of interests to declare.

## Author contributions

C. C. and A‐M. W. contributed to project design. C. C., X‐H. Z. and X‐C. W. performed the experiments and data analysis. J‐X. F. and H‐L. L presented technique supports. C. C., A‐M. W. and B. W. wrote the manuscript. A‐M. W. and J‐X. F. revised the article.
